# Management of alemtuzumab-induced Graves’ disease in pregnancy: a case report and literature review

**DOI:** 10.3389/fimmu.2025.1722459

**Published:** 2025-12-17

**Authors:** Marsida Teliti, Maria Gallo, Pietro Costa, Spyridon Chytiris, Flavia Magri, Mario Rotondi

**Affiliations:** 1 Department of Internal Medicine and Therapeutics, University of Pavia, Pavia, Italy; 2 Unit of Endocrinology and Metabolism, Laboratory for Endocrine Disruptors, Istituti Clinici Scientifici Maugeri IRCCS, Pavia, Italy

**Keywords:** alemtuzumab, pregnancy, ALZ-induced GD, multiple sclerosis, Graves’ disease

## Abstract

**Background:**

Alemtuzumab (ALZ), a monoclonal antibody used to treat relapsing-remitting multiple sclerosis (RRMS), is associated with a high risk of autoimmune thyroid disorders, particularly Graves’ disease (GD). Managing ALZ-induced GD during pregnancy presents unique challenges due to fluctuating thyroid function and potential fetal risks. However, the literature on this specific condition remains limited, with only a few case reports and commentaries available.

**Case report:**

The case-history of a 36-year-old woman diagnosed with GD at 11 weeks of gestation, 16 months after receiving her last ALZ dose is described. She was treated with methimazole (MMI), with multiple dose adjustments throughout pregnancy to maintain euthyroidism. Despite persistently elevated TSH receptor antibodies levels, fetal development occurred normally, and she delivered a healthy newborn. The infant experienced transient neonatal hyperthyroidism with spontaneous recovery without treatment. In the postpartum period, both mother and child were closely monitored. As the infant’s condition stabilized, the mother’s MMI dose was gradually increased. At seven months postpartum, she remains euthyroid on 15 mg/day of MMI, with no clinical/radiological signs of multiple sclerosis relapse.

**Conclusion:**

The present case adds to the limited literature on ALZ-induced GD in pregnancy, providing further insight into the variability of disease onset, progression, and neonatal outcomes. It underscores the importance of close monitoring and a multidisciplinary approach to ensure optimal maternal and fetal health.

## Introduction

Alemtuzumab (ALZ) is a humanized monoclonal antibody (anti-CD52 Ab) approved for the treatment of active relapsing-remitting multiple sclerosis (RRMS), widely used in Europe since the mid-1980s. In 2018, the U.S. Food and Drug Administration (FDA) issued a warning regarding rare but serious episodes of stroke in MS patients treated with ALZ (1). As a result, its use has been restricted since 2020, despite its positive effects on MS treatment (2–4). ALZ targets the cell-surface antigen CD52, which is expressed on the surface of more than 95% of T and B cells, monocytes, and some dendritic cells (5). ALZ induces cellular lysis and results in rapid, prolonged lymphocyte depletion (3, 4). After ALZ administration, circulating lymphocytes disappear within minutes. Within three months, B cells recover, with a predominance of mature naïve cells (CD19+ CD23+ CD27−) over memory B cells. Approximately 20 months later, CD8+ T cell counts are restored, and after 35 months, CD4+ T cell counts also recover (5).

Treatment with ALZ in patients with RRMS is associated with increased risk of immune-mediated conditions (1, 2). These adverse events encompass autoimmune thyroid disorders in up to 40% of cases, with Graves’ disease (GD) representing the most common thyroid dysfunction (~70%) (6–8). GD induced by ALZ often has a more unpredictable course compared to conventional GD, being typically characterized by fluctuating levels of stimulating and inhibitory TSH receptor antibodies (TRAb) (8). As a result, the clinical phenotype of GD can fluctuate between hyperthyroidism and hypothyroidism (8). These data suggest that immune reconstitution following ALZ treatment is primarily humoral, targeting the TSH receptor (TSH-R) as a major autoantigen (6).

Given that multiple sclerosis mainly affects women of reproductive age, the 2019 European Thyroid Association Guidelines on the Management of Thyroid Dysfunction following Immune Reconstitution Therapy recommend monthly thyroid function monitoring in women who are pregnant or planning pregnancy (9, 10). This proactive approach aims at early identification of thyroid dysfunction, enabling timely intervention. A multidisciplinary approach, involving neurologists, endocrinologists, and obstetricians, is crucial for the effective management of these complex cases, ensuring optimal maternal and fetal health (9, 10). The challenge of managing ALZ-induced GD is further compounded during pregnancy due to the dynamic changes in thyroid function and immune system that occur during gestation (11). In addition, transplacental TRAb transfer may expose the fetus and newborn to a significant risk of developing thyrotoxicosis (12–14).

At present, literature addressing the specific risks of ALZ-induced GD in pregnancy remains limited, with only a few case reports and commentaries (15–17).

We are here reporting the case of a patient who developed ALZ-induced GD early in pregnancy, emphasizing the complexity of its management and the value of a multidisciplinary approach to safeguard maternal and fetal outcomes. Additionally, we reviewed previously published cases to better define the course of this condition during pregnancy.

## Case presentation

A 36-year-old woman in the 11th week of her first pregnancy was referred to our Unit for new onset of hyperthyroidism. The patient had a history of multiple sclerosis, diagnosed at the age of 27, treated with ALZ administered in 3 courses at the ages of 27, 28 and 34 years. She had a positive family history for thyroid disorders. The patient denied smoking history. At presentation she reported fine distal tremors of the upper extremities. On physical examination, she had normal blood pressure and a heart rate of 120 beats per minute. The patient showed no signs of active ophthalmopathy. Her body weight was 58 kg and a height of 153 centimeters. Laboratory assessment (**Table 1**) showed increased free T4 of 27.3 pg/ml (nonpregnancy reference range 7-14.8 pg/ml) and a suppressed TSH (nonpregnancy reference range 0.47-4.78 mIU/L). Anti-thyroid peroxidase antibodies (TPOAb) and anti-thyroglobulin antibodies (TgAb) were negative. TRAb level was 20.6 IU/L (ref. range <1.75). Thyroid ultrasound (US) showed a normal-sized thyroid gland with a heterogeneous and pseudonodular echotexture and increased, uniform vascularization. Based on clinical and biochemical findings, a diagnosis of GD was rendered and methimazole (MMI) 10 mg/day was started. As treatment was initiated at 11 weeks of gestation, towards the end of the first trimester, when organogenesis is nearly complete and the 6–10-week teratogenic window has passed, MMI was chosen over PTU.

**Table 1:** Maternal thyroid function parameters, TRAb levels and treatment during pregnancy and post-partum.

Week of gestation	TSH mIU/L (0.47-4.78)	FT4 pg/ml (7-14.8)	FT3 pg/ml (1.58-3.91)	TRAb U/L (<1.75)	Treatment
11	0	27.3	13.78	20.6	MMI 10 mg
13	0	14.9	5.09	–	MMI 10 mg
16	0	11.1	4.83	–	MMI 10 mg
19	0	14.8	5.89	26	MMI 10 mg
21	0	16.4	6.27	–	MMI 15 mg
23	0	14.1	5.54	–	MMI 15 mg
25	0	11.9	4.71	37.9	MMI 15 mg
28	0	10.8	4.48	34.49	MMI 12.5 mg
30	0	13.0	3.49	35.9	MMI 12.5 mg
32	0	10.4	3.81	35.75	MMI 10 mg
34	–	–	–	–	MMI 10 mg
36	0	9.3	3.81	–	MMI 7.5 mg
38	0	14.4	3.92	35.3	MMI 7.5 mg
41	0	9.6	3.80	–	MMI 7.5 mg
1 month postpartum	0	14.6	3.17	48.5	MMI 10 mg
2 month postpartum	0	10.2	2.84	39.9	MMI 10 mg
4 months postpartum	0.20	10.0	2.87	32.7	MMI 15 mg
5 months postpartum	4.55	10.5	3.15	18.8	MMI 15 mg
7 months postpartum	2.08	10.6	2.57	30	MMI 15 mg

TSH, thyroid-stimulating hormone; fT4, free thyroxine; fT3, free triiodothyronine; TRAb, TSH receptor antibodies; MMI, methimazole – indicates data not available or not assessed at that timepoint.

Subsequently, thyroid function tests were performed every 2/3 weeks. Until the 19th week of gestation, the patient continued on the same dose of MMI. At 21 weeks’ gestation, because of a progressive increase of FT3 and FT4, MMI dose was increased to 15 mg/day. At 25 weeks’ gestation, TRAb remained elevated at 20.6 IU/L (reference range <1.75), with no significant decline over time. Over the following weeks, free thyroid hormone levels decreased. At 28 weeks of gestation, MMI was reduced to 12.5 mg/day in response to the gradual decline in circulating FT4 levels. Later, at 32 weeks, the dose was further lowered to 10 mg/day and subsequently titrated down to 7.5 mg daily at 36 weeks’ gestation. This dose was maintained until the end of pregnancy (**Table 1**).

### Postpartum follow-up and current status

The MMI dose was gradually increased thereafter. Currently, at seven months postpartum, the patient is euthyroid on 15 mg/day of MMI (**Table 1**) and shows no clinical or radiological signs of MS relapse, with postpartum MRI and follow-up confirming disease stability.

**Figure 1:**
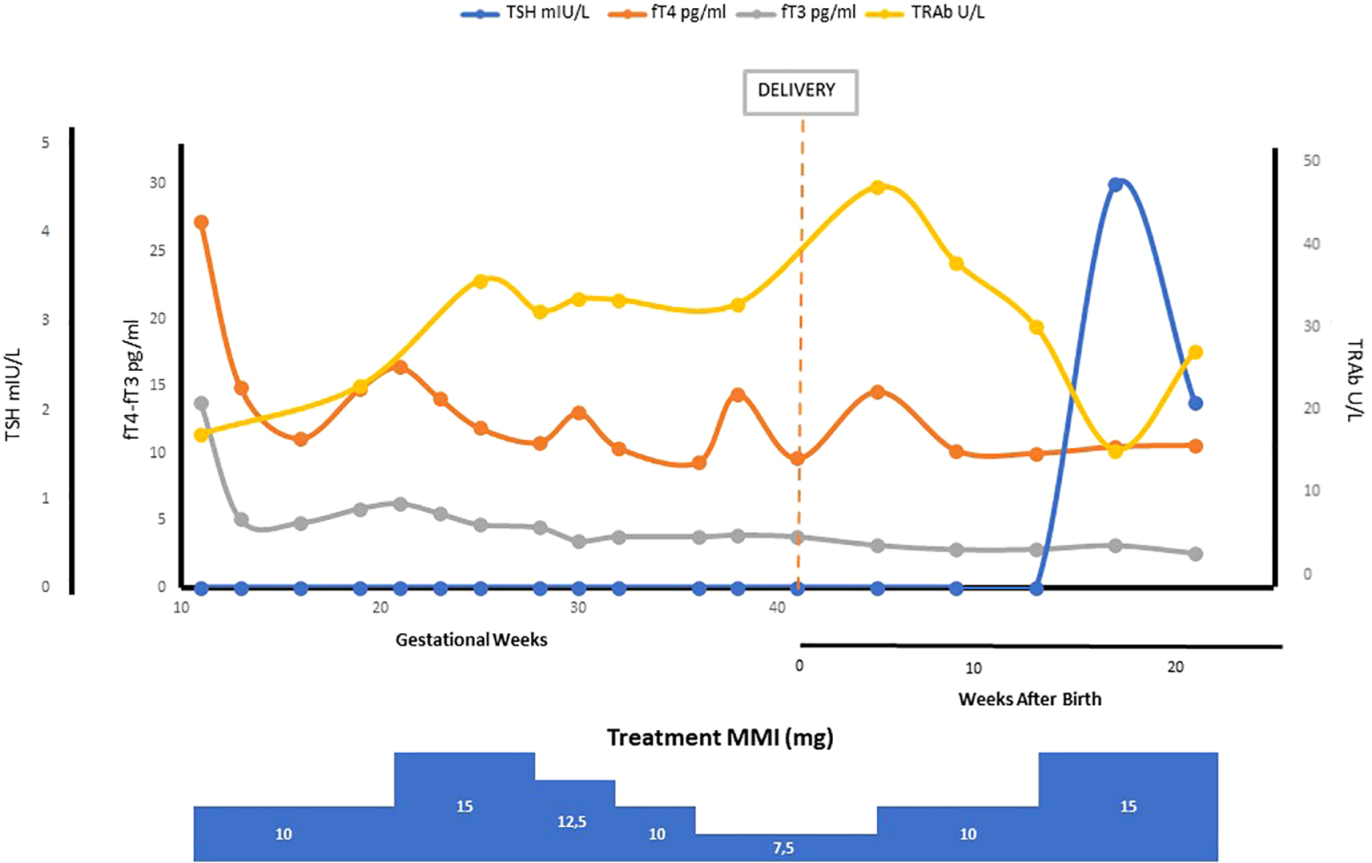
Maternal thyroid function parameters, TRAb levels and medications during pregnancy and postpartum.

Changes in thyroid function parameters and TRAb throughout gestation and postpartum, in relation with timing and maternal treatment are shown in **Figure 1**.

### Fetal and neonatal outcome

During pregnancy regular obstetric US showed normal fetal growth and no signs of fetal hyperthyroidism. The patient delivered a baby girl weighing 3610g without obstetric complications. At birth, a normal Apgar score was reported. Neonatal thyroid testing revealed an euthyroid status with high-titer TRAb (28.2 IU/L, normal <0.55 IU/L, **Table 2**). At 10 days of life, thyroid function tests indicated neonatal hyperthyroidism, despite a decline in TRAb levels. The infant was admitted to the hospital for further evaluation. Her thyroid US, physical examination, electrocardiography, and growth rate were all normal. Owing to the progressive amelioration of thyroid function, no antithyroid therapy was initiated.

**Table 2:** Neonatal thyroid function parameters and TRAb levels.

Age	TSH mIU/L (0.4-4.0)	FT4 pg/ml (8-19)	FT3 pg/ml (1.8-4.2)	TRAb UI/L (<0.55)
3 days	–	–	–	28.2
10 days	0.048	28.84	8.65	8.71
15 days	0.037	14.98	4.67	–
19 days	0.714	12.96	4.70	1.94
24 days	3.982	10.79	4.79	1.18
45 days	9.811	9.63	4.44	–
75 days	3.269	13.31	4.75	0.45

TSH, thyroid-stimulating hormone; fT4, free thyroxine; fT3, free triiodothyronine; TRAb, TSH receptor antibodies – indicates data not available or not assessed at that timepoint.

## Discussion and comparison with literature

We report a successful pregnancy in an ALZ-treated patient who developed GD hyperthyroidism with a high TRAb titer at the end of the first trimester, requiring ATD treatment. Our case highlights the complexities of managing ALZ-induced GD during pregnancy, a condition that remains yet to be fully elucidated in view of the limited available evidences (15–17). To date, only three cases of ALZ-induced GD during pregnancy have been reported. **Table 3** compares these reports with the present case, highlighting key differences in disease onset, TRAb levels, and maternal–neonatal outcomes (15–17).

**Table 3:** Key differences in disease onset, TRAb levels, and maternal-neonatal outcomes of the here presented case with the three previously reported in literature.

Feature	Present case	Case 1 (15)	Case 2 (16)	Case 3 (17)
Patient’s Age	36 years old	31 years old	36 years old	27 years old
GD Onset	First trimester (11 weeks)	First trimester (5 weeks)	First trimester (12 weeks)	Late third trimester (28 + 3 weeks)
ALZ Interval	16 months post-ALZ	32 months post-ALZ	15 months post-ALZ	22 months post-ALZ
TRAb at Diagnosis	20.6 IU/L	2.94 IU/L	15.2 IU/L	>40 IU/L
TRAb Progression	Increased >35 IU/L in third trimester	Declined to 0.859 IU/L before delivery	Increased >40 IU/L in third trimester	Persistently >40 IU/L
Maternal ATD Treatment	MMI (7.5–15 mg/day, titrated)	PTU (first trimester), then CBZ 5 mg/day (weeks 18-25)	CBZ 60 mg/day, increased in third trimester	High-dose PTU (200–300 mg/day)
Fetal Thyrotoxicosis	None (serial US normal)	None (serial US normal)	Detected	Not detected *in utero* (serial US normal)
Neonatal Thyrotoxicosis	Delayed onset at day 10, requiring hospitalization	Not present (maternal TRAb declined before delivery)	Transient, treated at birth	Severe, requiring treatment from day 3 to 3 months
Post-partum Course	Infant required close monitoring, maternal ATD tapered	Maternal GD relapse at 11 months postpartum	Persistent maternal dysfunction, thyroidectomy referral	Prolonged neonatal hyperthyroidism (CBZ until 3 months)

GD, Graves' disease; ALZ, Alemtuzumab; TRAb, TSH receptor antibodies; ATD, Antithyroid Drugs, MMI, Methimazole, PTU, Propylthiouracil, CBZ, Carbimazole.

As to timing of occurrence of ALZ-induced GD in our case, the patient developed GD 16 months after receiving her third course of ALZ, similar to the case reported by Garrahy et al. (16). In contrast, the other two patients developed ALZ-induced GD in pregnancy 32 months and 22 months after their last doses of ALZ, respectively (15, 17).

From a clinical point of view, the present findings align with previous reports of ALZ-induced GD in pregnancy, particularly those by Garrahy et al. and Thakar et al. (16, 17), which described aggressive disease courses with rising TRAb titers throughout gestation requiring continued ATD therapy and neonatal intervention. At difference, Kazakou et al., reported a milder case where TRAb levels declined in late pregnancy, leading to spontaneous resolution before delivery (15).

Review of the above cases would suggest that when ALZ-induced GD occurs in early pregnancy, disease severity varies, with some cases resolving spontaneously (15), and others requiring escalating ATD therapy (16). Moreover, these cases illustrate the broad spectrum of the neonatal repercussions of maternal ALZ-induced GD, ranging from mild-transient hyperthyroidism (15) to severe neonatal thyrotoxicosis (17). Although fetal thyrotoxicosis was reported only once (17), neonatal hyperthyroidism occurred in three cases, two of whom required treatment with ATDs (16, 17).

Typically in nonpregnant women, thyroid immune-related adverse reactions have a delayed and variable onset, occurring 6–23 months after the last ALZ dose (8). Accordingly, guidelines for patients outside pregnancy recommend regular biochemical surveillance, including TSH testing every three months. Thyroid function monitoring should continue for at least four years from the last ALZ treatment. Beyond this period, TSH testing should be based on symptoms or clinical suspicion of thyroid dysfunction. Routine monitoring of thyroid autoantibodies is not recommended (10).

However, there is a lack of specific guidance for pregnancy. Current prescribing guidelines recommend that women of childbearing potential use effective contraception for four months after receiving ALZ (18).

During pregnancy, thyroid function can undergo significant changes, increasing the risk of thyroid dysfunction for both the mother and the fetus. Therefore, it is advisable to intensify monitoring during pregnancy, with monthly assessments of TSH to ensure timely intervention in case of abnormalities (11). Pregnancy necessitates a more intensive monitoring approach compared to standard guidelines for patients treated with ALZ to ensure both maternal and fetal health (8).

The comparative analysis of these four cases provides valuable insights into the clinical course of ALZ-induced Graves’ disease (GD) in pregnancy. These cases collectively demonstrate the unpredictable nature of ALZ-induced GD in pregnancy, emphasizing the need for a personalized, multidisciplinary approach to optimize maternal and fetal outcomes. Key recommendations include frequent TRAb monitoring, individualized ATD therapy, close fetal surveillance, and extended postpartum follow-up for both mother and child (8). Although informative, these observations must be interpreted with caution, as they are based on a very small number of cases (n=4), which limits the generalizability of the conclusions and highlights the need for further evidence.

## Conclusions

ALZ remains a highly effective yet complex treatment for RRMS, with autoimmune thyroid dysfunction, particularly GD, being the most frequent adverse effects (7, 8). ALZ-induced GD presents unique challenges, especially in pregnant patients, where careful management is crucial to ensuring both maternal and fetal well-being.

The present clinical case contributes to the limited reports in the literature on this topic and provides further evidence on the variability of disease onset, progression, and neonatal outcomes.

Given that ALZ is frequently prescribed to young women in childbearing age (19, 20), establishing standardized guidelines for reproductive-age patients is mandatory to improve outcomes and ensure safer pregnancies.

## Data Availability

The raw data supporting the conclusions of this article will be made available by the authors, without undue reservation.
